# Mitochondrial DNA data allow distinguishing the subpopulations in the widespread Demoiselle crane (Anthropoides virgo)

**DOI:** 10.18699/vjgb-25-60

**Published:** 2025-07

**Authors:** Е.А. Mudrik, Е.I. Ilyashenko, P.A. Kazimirov, K.D. Kondrakova, T.P. Archimaeva, L.D. Bazarov, О.А. Goroshko, Ts.Z. Dorzhiev, A.N. Kuksin, К.А. Postelnykh, V.V. Shurkina, V.Yu. Ilyashenko, A.V. Shatokhina, D.V. Politov

**Affiliations:** Vavilov Institute of General Genetics of the Russian Academy of Sciences, Moscow, Russia; Vavilov Institute of General Genetics of the Russian Academy of Sciences, Moscow, Russia Severtsov Institute of Ecology and Evolution of the Russian Academy of Sciences, Moscow, Russia; Vavilov Institute of General Genetics of the Russian Academy of Sciences, Moscow, Russia; Vavilov Institute of General Genetics of the Russian Academy of Sciences, Moscow, Russia Severtsov Institute of Ecology and Evolution of the Russian Academy of Sciences, Moscow, Russia; Tuvinian Institute for Exploration of Natural Resources of the Siberian Branch of the Russian Academy of Sciences, Kyzyl, Russia; Tunkinsky National Park, Kyren, Russia; Institute of Nature Resources, Ecology and Cryology of the Siberian Branch of the Russian Academy of Sciences, Chita, Russia Daursky State Nature Biosphere Reserve, Nizhny Tsasuchey, Russia; Buryat State University named after Dorji Banzarov, Ulan-Ude, Russia Institute of General and Experimental Biology of the Siberian Branch of the Russian Academy of Sciences, Ulan-Ude, Russia; Tuvinian Institute for Exploration of Natural Resources of the Siberian Branch of the Russian Academy of Sciences, Kyzyl, Russia; Oka State Nature Biosphere Reserve, Brykin Bor, Russia; Khakassky State Nature Reserve, Abakan, Russia; Severtsov Institute of Ecology and Evolution of the Russian Academy of Sciences, Moscow, Russia; Vavilov Institute of General Genetics of the Russian Academy of Sciences, Moscow, Russia; Vavilov Institute of General Genetics of the Russian Academy of Sciences, Moscow, Russia

**Keywords:** Gruidae, gene pool, cytochrome b (cyt b), haplotype, genetic diversity, genetic differentiation, population-genetic structure, flywayFor, Gruidae, генофонд;, цитохром b, гаплотип, генетическое разнообразие, генетическая дифференциация, популяционно-генетическая структура, пролетный путь

## Abstract

The polymorphism of the mtDNA cytochrome b (cyt b) gene’s partial sequences has been studied in the Demoiselle crane (Anthropoides virgo Linnaeus, 1778) for the first time. Based on cyt b variability, the population genetic structure of the species was characterized within most of its range in Russia. Among 157 individuals we identified 18 haplotypes, nine of which were unique. In the European samples, we observed greater haplotype and nucleotide diversity and stronger genetic differentiation than in the Asian ones. Gene flow between different parts of the Demoiselle crane range is probably mediated by birds breeding in the Trans-Urals. The overall genetic subdivision of the species as estimated by FST was 0.265 (p <0.001). The structure of the gene pool is formed by three main haplotypes, one of which predominates in the Azov-Black Sea region, the second in the Caspian and Volga-Ural regions, and the third is most common in the Asian samples. Based on the correspondence of intraspecific genetic differentiation of the Demoiselle cranes from different parts of the range to their flyways, we propose to distinguish the following subpopulations: (1) Azov-Black Sea/Chadian; (2) Caspian/Sudanese; (3) Trans-Ural/Indian; (4) South Siberian/Indian; (5) Baikal/Indian and (6) Trans-Baikal/Indian. The obtained data create the basis for monitoring the genetic diversity of the Demoiselle crane and developing a scientific background for measures to protect the gene pool of the species as a whole and its subpopulations.

## Abstract (ru)

Впервые изучен полиморфизм последовательностей фрагмента гена мтДНК цитохрома b и на его основе охарактеризована популяционно-генетическая структура журавля красавки (Anthropoides virgo Linnaeus, 1778) на большей части ареала в России. Для 157 особей идентифицировано 18 гаплотипов, девять из которых оказались уникальными. Европейские выборки характеризовались большей гаплотипической и нуклеотидной изменчивостью и более сильной генетической дифференциацией, чем азиатские. Поток генов между разными частями ареала красавки, вероятно, осуществляется через птиц, гнездящихся в Зауралье. Общая генетическая дифференциация вида составила >20 % (FST = 0.265, p < 0.001). Структура генофонда сформирована тремя основными гаплотипами, один из которых преобладает в Азово-Черноморском регионе, второй – в Прикаспийском и Волго-Уральском, а третий наиболее распространен в азиатских выборках. Исходя из соответствия внутривидовой генетической дифференциации красавки пролетным путям птиц из разных частей ареала, мы предлагаем в структуре вида выделить следующие субпопуляции: 1) азово-черноморско-чадскую; 2) прикаспийско-суданскую; 3) зауральско-индийскую; 4) южносибирско-индийскую; 5) байкальско-индийскую; 6) забайкальско-индийскую. Полученные данные создают основу для мониторинга генетического разнообразия красавки и разработки научного обоснования мер охраны генофонда как вида в целом, так и его отдельных субпопуляций.

## Introduction

The Demoiselle crane (Anthropoides virgo, Linneaus, 1778) is a Eurasian crane species, the gene pool of which has been studied only fragmentarily. The breeding part of the Demoiselle crane range extends across the steppe and semi-desert zones from the Azov-Black Sea region of Russia eastward to North-Eastern China. In Europe, the species is categorized as endangered (BirdLife International, 2021) due to habitat degradation, periods of prolonged drought, and a steady population number decline caused, among other things, by hunting at migration routes and wintering grounds, while on a global scale its status is evaluated as least concern (BirdLife International, 2018). The Demoiselle crane is listed in the Red Book of the Russian Federation (Ilyashenko, 2021). The remaining European breeding groups are almost completely localized in the territory of the south of the European part of Russia. As for the Asian part of the range, the core of which is centered in Kazakhstan and Mongolia, its northern border runs along the southern regions of the Trans-Urals and Siberia (Ilyashenko, 2019).

The first and only data on the population genetic structure of the Demoiselle crane to date were obtained by us using microsatellite loci and sequences of the Control Region (CR) of mtDNA. A high level of genetic diversity was revealed for both types of markers in all parts of the range. European groupings have been shown to be more subdivided than Asian ones, and in general, the genetic differentiation of the species is low (Mudrik et al., 2018, 2022). However, these studies were carried out on a small number of individuals from the wild, especially as far as the Asian part of the range is concerned. In addition, some biomaterial from zoo birds was also included. At the same time, the analysis of the CR, justified by the high variability of this non-coding region of mtDNA, may not reflect the structure of the gene pool formed by proteincoding genes, among which cytochrome b is recognized as one of the most reliable markers (Zardoya, Meyer, 1996). The overwhelming majority of population genetic studies of cranes were performed on the CR, while information on the polymorphism of the sequences of the more conservative cytochrome b in this group of birds is quite scarce (we found only a few identical Demoiselle crane cyt b sequences in the NCBI Genbank). In this regard, we set the goal of assessing for the first time at the population level and on a large geographic scale the polymorphism of the mitochondrial cytochrome b gene in the Demoiselle crane and characterizing its gene pool in different parts of the range using more representative biomaterial from nature than in previous studies, primarily from previously unstudied Asian groups.

## Materials and methods

Biological sample collection. In this research, we used biological specimens from 157 individuals of the Demoiselle crane. The study was approved by the Local Bioethics Committee at the Vavilov Institute of General Genetics of the Russian Academy of Sciences (protocols No. 1 dated May 15, 2017 and No. 1 dated May 18, 2023). The source of DNA was blood (or, less often, epidermis) from plucked feathers from the chest or neck area of chicks aged 15–35 days. Feather samples were collected in 2016–2024 during our own expeditions to Demoiselle crane breeding locations run during the seasons when the chicks were yet unable to fly (June–July). The chicks were caught by hand in accordance with permits from the Federal Service for Supervision of Natural Resources of the Russian Federation No. 43 (2016); No. 104, 105, 106 (2017); No. 52, 56 (2018); No. 9, 60 (2019); No. 21 (2023); and No. 78 (2024). After collecting the biomaterial and tagging, which usually takes 5–10 min, the chicks were released and monitored until they rejoined their parents. Plucked feathers were placed in Longmire’s preservative solution in screw tubes, transported to the laboratory at room temperature, and then stored in a freezer at –20 ºC. Normally, a Demoiselle crane brood consists of two chicks, and if biomaterial from both sibs was available, a specimen from only one sibling of each pair was included in the analysis.

To designate samples in the European part of the range, we followed the established division into breeding groups (Belik et al., 2011), while for the Asian part, we assigned topographic names to the samples. So, we analyzed 156 unrelated individuals from 10 samples covering most of the species range in Russia: Azov-Black Sea, Caspian, Volga-Ural (which includes several individuals from Western Kazakhstan), Cis-Ural (European part); Trans-Ural, Khakass, Altai, Tyvan, Baikal and Trans-Baikal (Asian part). Some samples (Cis-Ural, Trans-Ural, Khakass, Altai) were represented by a small but the maximum available number of birds due to the low density of Demoiselle cranes in the corresponding study areas and/or low success of their reproduction during the years of field work. In order to increase the Altai sample, we additionally sequenced the biomaterial of an individual kept in the Barnaul Zoo, which, according to documents, originated from the nature of the Altai Krai.

Molecular genetic analysis. Genomic DNA was extracted from plucked feathers using the K-sorb kit (Syntol, Russia) according to the manufacturer’s protocol. Amplification of the cytochrome b gene fragment was carried out using forward (F: CTACTACTAGCYGCACACTA) and reverse (R: AGG TTGGCGGTTAGGGTTC) oligonucleotide primers (Sun et al., 2020) and the GenPak PCR Core Reagent kit (Isogen Laboratory LTD, Russia) on a GeneExplorer amplifier, model GE-96G (Bioer Technology Co LTD, China). The amplification program consisted of pre-denaturation (94 °C for 5 min), 30 cycles (94 °C for 30 s, 55 °C for 30 s, 72 °C for 1 min) and final elongation (72 °C for 10 min) (Sun et al., 2020). The size and quality of the amplification products were checked by electrophoresis in 1.5 % agarose gel, then they were purified using Cleanup St PCR kits (Evrogen, Russia) and sequenced in the forward direction on an ABI 3130 Genetic Analyzer (Applied Biosystems, USA) at Evrogen Joint Stock Company (Russia).

Analysis of molecular genetic data. Alignment of approximately 900 bp cytochrome b sequences obtained from Sanger sequencing was performed against each other and the only complete sequence of this gene of the Demoiselle crane in NCBI Genbank (NC_020573) using the MAFFT algorithm (Katoh et al., 2002) in Geneious v. 9.1.8 (Kearse et al., 2012). Nucleotide diversity, selective neutrality tests, pairwise and total estimates of genetic subdivision of GST and FST, female gene flow (number of female migrants per generation) Nm were calculated using DnaSP v. 6.11.01 (Librado, Rozas, 2009). AMOVA analysis of molecular variability and construction of the median haplotype network using the TCS algorithm (Clement et al., 2002) were performed in PopART (Leigh, Bryant, 2015). Maximum Likelihood haplotype trees were constructed using the IQTree service (Trifinopoulos et al., 2016; Kalyaanamoorthy et al., 2017; Minh et al., 2020) based on the HKY+F (Hasegawa–Kishino–Yano) nucleotide substitution model (Hasegawa et al., 1985), selected as optimal according to the Bayesian criterion (BIC). Branch node support was calculated using the UltraFast Bootstrap method for 1,000 replications (Hoang et al., 2017). The cytochrome b sequence of the Demoiselle crane’s closest relative, the Blue crane (Anthropoides paradiseus) (Genbank accession number U27557), was used as an outgroup.

Graphical visualization of trees was performed in the R environment (R Core Team, 2022) using the ggtree (Yu et al., 2017, 2018; Yu, 2020, 2022), ggtreeExtra (Xu et al., 2021; Yu, 2022), tidytree (Yu, 2022), ggplot2 (Wickham, 2016), pals (Wright, 2024), and ggnewscale (Campitelli, 2024) packages. A heat map of haplotype similarity based on nucleotide substitutions was constructed in the R environment using the algorithm described in the article (Toparslan et al., 2020). To create maps with the geographic localization of haplotypes, the following packages were used: ggmap (Kahle, Wickham, 2013), ggrepel (Slowikowski, 2024), smoothr (Strimas-Mackey, 2023), sp (Pebesma, Bivand, 2005; Bivand et al., 2013) and pals, as well as basic methods of the R environment.

## Results

**distribution patterns**

**Fig. 1.**
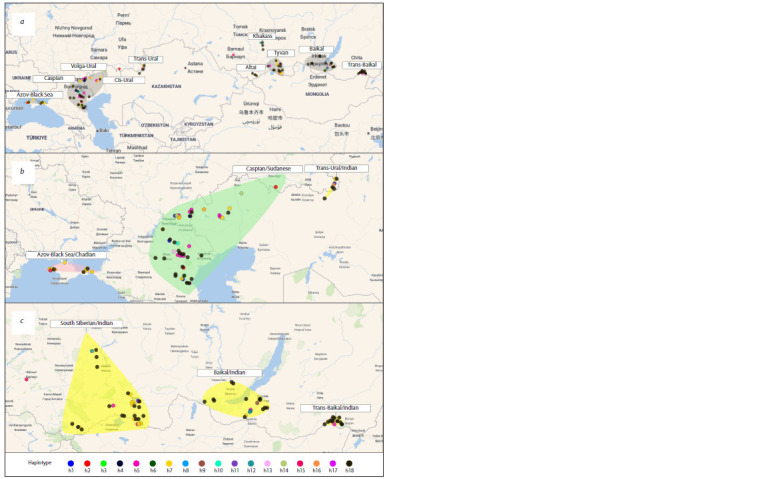
Location of the Demoiselle crane cytochrome b haplotypes in the studied samples (a) and subpopulations identified in the European (b) and Asian (b, c) parts of the range. The contours of the subpopulations are conditional, since they only outline the points of material collection.

After alignment, the size of the analyzed sequences was 771 bp. In the total sample of 157 birds, 18 haplotypes were identified (Table 1, Fig. 1a) (Genbank accession numbers PQ663762–PQ663779). Nine of them (h1, h2, h3, h5, h7, h12, h14, h15, h18) were found in at least two breeding groups. The most frequent (in 50.9 % of individuals) was haplotype h18; it was present in all samples except Cis-Ural. Also, haplotypes h7 (except Cis-Ural and Khakass, 23.6 % of individuals) and h5 (except Azov-Black Sea and Altai, 11.5 % of individuals) were spread almost throughout the entire range. Unique haplotypes were found in the Caspian (h6, h10, h13), Trans-Ural (h4, h16), Tyvan (h8, h17), Baikal (h9) and Trans- Baikal (h11) samples. No unique haplotypes were found in the Azov-Black Sea and Volga-Ural samples; however, the most frequent ones were h7 and h5, respectively, but not h18, as in the others. The only individual from the Cis-Ural sample had a non-unique haplotype: the same was present in the Caspian sample (h2). The haplotype of the Demoiselle crane from the Barnaul Zoo turned out to be the same as in the Azov-Black Sea region (h15) (Table 1), and since the reliability of the origin of this bird was not obvious, we excluded it from the subsequent population genetic analysis, as well as the only available Cis-Ural individual.

**Table 1.**
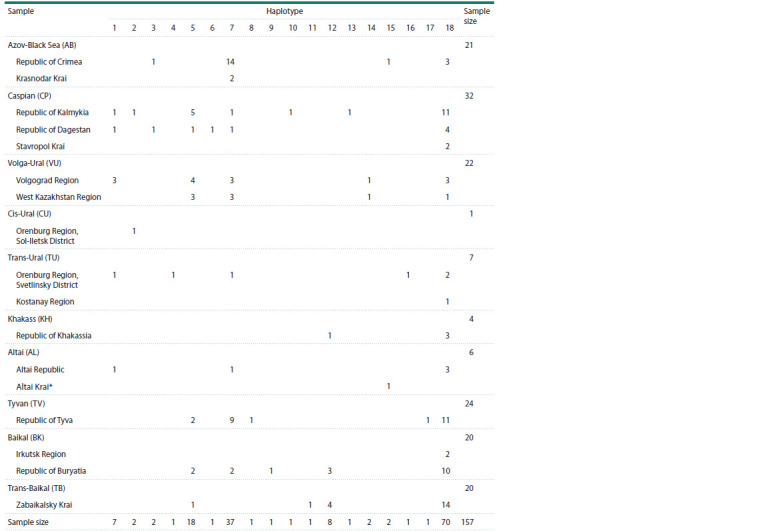
Distribution of cytochrome b haplotypes in the Demoiselle crane samples * Bird from the Barnaul Zoo, presumably from the Altai Krai.

**Genetic diversity and differentiation**

In general, the European and Asian samples were comparable in the number of individuals analyzed, and the same number of haplotypes (11) and segregating sites (10) of cytochrome b were found in them (Table 2). The samples from the western (Azov-Black Sea) and eastern (Trans-Baikal) boundaries of the range showed the lowest haplotype (Hd) and nucleotide (π) diversity compared to other samples and the average values for Europe and Asia and the species as a whole. Reduced values of these indices compared to the average were also found in the Khakass sample, which was the northernmost of those studied.

**Table 2.**
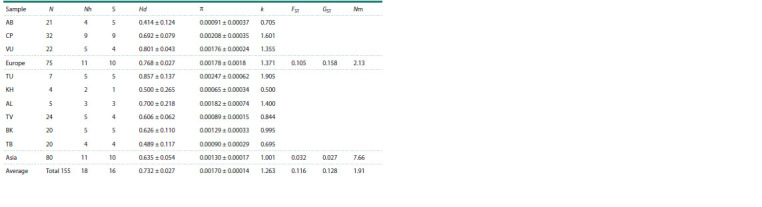
Summary statistics of polymorphism and genetic differentiation of the Demoiselle crane samples according to cytochrome b data Notе. N – sample size; Nh – haplotype number; S – segregating site number; Hd – haplotype diversity; π – nucleotide diversity; k – average number of nucleotide differences; FST and GST – genetic subdivision estimates; Nm – gene flow. Samples: AB – Azov-Black Sea, CP – Caspian, VU – Volga-Ural, CU – Cis-Ural, TU – Trans- Ural, KH – Khakass, AL – Altai, TV – Tyvan, BK – Baikal, TB – Trans-Baikal.

The maximal number of haplotypes (9) was found in the Caspian sample, while the Volga-Ural and Trans-Ural samples had the highest haplotype diversity. In general, the values of haplotype and nucleotide diversity indices as well as the average number of nucleotide differences were higher in the European part of the range (Hd = 0.768 ± 0.027; π = 0.00178 ± 00018; k = 1.371) compared to the Asian part (Hd = 0.635 ± 0.054; π = 0.00130 ± 0.00017; k = 1.001).

**Analysis of the similarity and spatial distribution of**

**Fig. 2.**
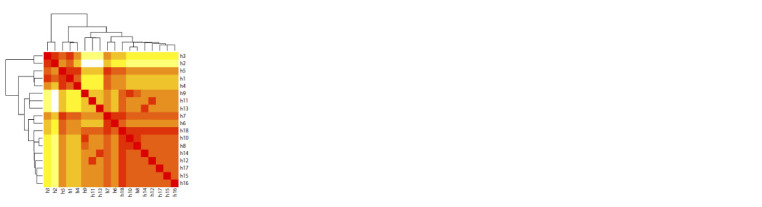
Heat map of nucleotide differences between Demoiselle crane cytochrome b haplotypes. Color intensity indicates degree of similarity (decreasing from darkest to lightest tint).

**Fig. 3.**
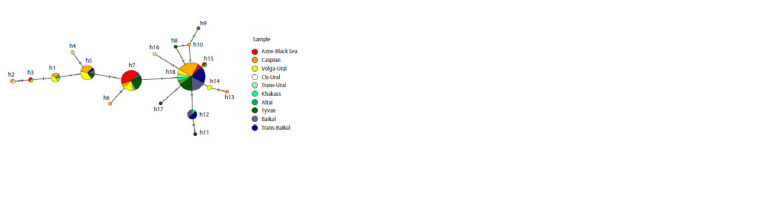
Median network of Demoiselle crane cytochrome b haplotypes constructed using the TCS algorithm. The circle size is proportional to the number of individuals, the length of the branches corresponds to the genetic distances, the notches indicate the number of mutation events, and the pie charts display the frequencies of haplotypes in the samples.

The heat map of haplotype similarity showed two clusters (h1–h5 and h6–h18), within which haplotypes h5, h7, h6, and h18 demonstrated the greatest similarity to haplotypes from outside of their own group (Fig. 2). This is probably due to the fact that h5, h7, and h18 were the most widespread haplotypes, occurring in almost all samples from the studied part of the range of the Demoiselle crane. On the median network, haplotype h7 (and its derivative h6) was located between h5 and h18 (Fig. 3).

**Fig. 4.**
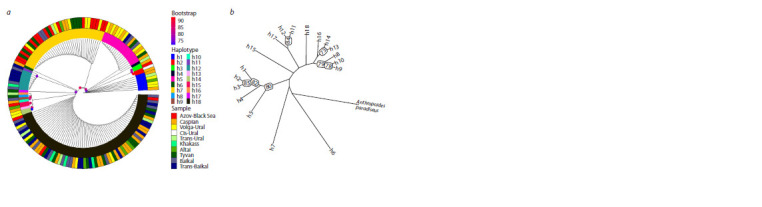
Maximum likelihood clustering (ML-trees) of individuals (a) and haplotypes (b) of the Demoiselle crane by nucleotide sequences of cytochrome b. In the left figure, the outer circle illustrates the belonging of individuals to samples, the inner one illustrates the belonging of individuals to haplotypes.

The cluster formed by h5 included haplotypes of European samples and the geographically close Trans-Ural sample, and the cluster in which the central haplotype was h18 was distributed throughout the entire studied part of the species range. This is also confirmed by the clustering of individuals on the ML-tree, which demonstrates the intermediate position of individuals with the h7 haplotype relative to h5 and h18 with a high degree of bootstrap support (Fig. 4a).

The tree constructed using the outgroup indicated that the cytochrome b haplotypes did not form a single monophyletic group, and the h7 haplotype was putatively ancestral to the other two most frequent haplotypes, h5 and h18, and their derivatives (Fig. 4b).

**Genetic differentiation and gene flow**

Genetic differences between the studied samples generally reflected their relative geographic location. The highest genetic differences were found between the most distant Azov-Black Sea and Trans-Baikal (FST = 0.4675), as well as between the northernmost Khakass and two European samples – Azov- Black Sea and Volga-Ural (Table 3). There was no genetic differentiation detected between the most geographically close Volga-Ural and Trans-Ural; Altai and Tyvan; Baikal and Trans- Baikal groups, as well as between some other samples within the European and Asian parts of the range. The Trans-Ural sample, geographically close to the European ones, but belonging to the Asian group, was genetically indiscernible from the Caspian one in Europe and the Altai and Tyvan ones in Asia, and differed only slightly from all other studied samples, which was probably due to its westernmost position in the Asian part of the range. Genetic subdivision within the European samples (FST = 0.105, GST = 0.158) was more pronounced than that among the Asian ones (FST = 0.032, GST = 0.027), which was putatively associated with a more limited gene flow in Europe (Nm = 2.13) compared to Asia (Nm = 7.66) (Table 2). The average values of these parameters for the species were estimated as: FST = 0.116, GST = 0.128, Nm = 1.91. Selective neutrality test values for cytochrome b were slightly negative and statistically insignificant (D = –1.514, F = –1.618), indicating the absence of the Demoiselle crane population expansion in the recent evolutionary past, just as we have shown previously for the CR sequence analysis (Mudrik et al., 2018, 2022).

**Table 3.**
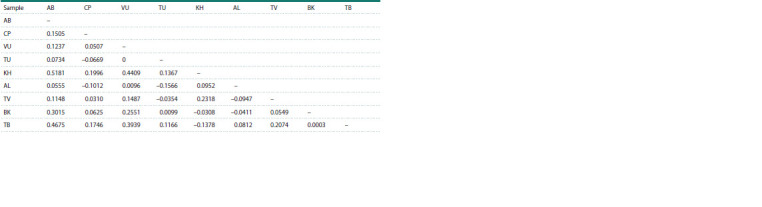
Pairwise values of genetic subdivision statistic FST between the Demoiselle crane samples based on cytochrome b sequence data Notе. AB – Azov-Black Sea, CP – Caspian, VU – Volga-Ural, CU – Cis-Ural, TU – Trans-Ural, KH – Khakass, AL – Altai, TV – Tyvan, BK – Baikal, TB – Trans-Baikal samples.

Hierarchical analysis of molecular variance AMOVA showed that the genetic differentiation of the total studied sample of the Demoiselle crane was 18.57 % (Level I: FST = 0.1875, p < 0.001), and when divided into European and Asian groups, it was 26.52 % (Level II: FST = 0.26524, p <0.001) (Table 4).

**Table 4.**
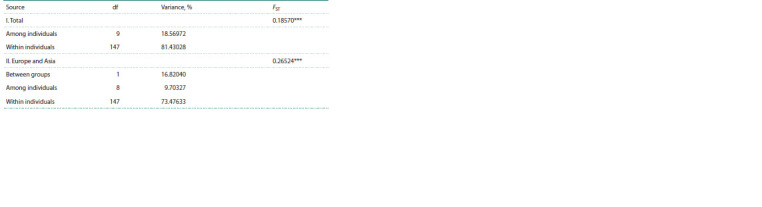
Results of AMOVA in the total sample of the Demoiselle crane and when divided into European and Asian groups according to cytochrome b data Notе. df – number of degrees of freedom; *** p <0.001.

## Discussion

Analysis of the nucleotide sequences of cytochrome b in the Demoiselle crane on a large geographical scale and a representative sample of birds from nature revealed polymorphism of this gene and a more pronounced population genetic structuring of the species compared to the data obtained previously for the Control Region of mtDNA (Mudrik et al., 2018, 2022). The lowest haplotype and nucleotide diversity were found in the westernmost (Azov-Black Sea), easternmost (Trans-Baikal) and northernmost (Khakass) samples, which was probably due to their attribution to marginal populations surviving at the edges of the species range. The highest values of these parameters were found eastwards of the Volga River and on both sides of the Urals, i. e. in the Volga-Ural and Trans-Ural samples. The largest number of haplotypes, including unique ones, was found in the Caspian region.

The cytochrome b gene pool structure of the Demoiselle crane was formed by the three most frequent haplotypes h5, h7 and h18, of which the most frequent haplotype from the Azov-Black Sea region, h7, was presumably ancestral. It is interesting that the Azov-Black Sea birds differ from other European and especially Asian ones in their migration routes over the Black and Mediterranean Seas and their wintering ground in the Republic of Chad at the junction of North and Central Africa, which was recently discovered using GPSGSM telemetry (Ilyashenko et al., 2021). Probably, such isolation and greater similarity to the outgroup (the related African species Blue crane) compared to other haplotypes has an evolutionary basis, which needs to be studied further using genomic methods.

The most frequent haplotype in the total studied sample, h18, found in more than half of the individuals and equally common with higher frequencies in the most remote Asian samples (Tyva, Buryatia and Transbaikalia) and prevailing in Altai and Khakassia (Fig. 1a), formed a “star” from which most other cytochrome b haplotypes, including unique ones, originated (Fig. 3). Birds from all these samples use a common wintering ground in the states of Rajasthan and Gujarat in India, and most of them (except for the Trans-Ural ones) make loop migrations, crossing the Himalayas in autumn and skirting the Tien Shan from the west in spring, sharing a significant part of the flyway (Ilyashenko et al., 2021). All this putatively contributes to gene flow among local breeding groups and a decrease in genetic subdivision of Demoiselle cranes in this part of the range. Genetic differences between Trans-Baikal and Baikal; Altai and Tyvan; Baikal, Altai and Khakass samples were practically absent (Table 3).

Finally, the third of the above-mentioned structure-forming haplotypes h5, lying on the median network on the other side than h18 from the central haplotype h7, was the most frequent in the Trans-Volga region (Volga-Ural sample) and formed the branch of “European” haplotypes, which also included haplotypes from the Cis-Ural and Trans-Ural samples. It should be noted that the previously identified Volga-Ural and Caspian breeding groups (Belik et al., 2011) are essentially a single genetically homogeneous (Table 3, Fig. 1b) subpopulation using common migration routes over the Arabian Peninsula and the Red Sea to wintering grounds in Sudan and partly Ethiopia in Africa (Ilyashenko et al., 2021). The only individual from the Cis-Ural sample had the same haplotype as the bird from Kalmykia (Caspian sample) (Table 1) and used the same flyway and pre-migratory gathering site in the Manych Valley as the Caspian and Volga-Ural cranes (Ilyashenko et al., 2021, 2024), which allows it to be classified as part of this subpopulation. It is noteworthy that in the “European” group of haplotypes, half of the haplotypes from the Trans-Ural sample are present. Although the Trans-Ural sample is geographically close to the European ones in the breeding part of the range (Fig. 1a), it uses wintering site in India, like all other Asian Demoiselle cranes. However, birds from the Trans-Ural sample fly in both autumn and spring through Kazakhstan, Uzbekistan, Tajikistan and Pakistan, without making a loop migration (Ilyashenko et al., 2021). According to the FST values, the Trans-Ural cranes have no genetic differences from the Volga-Ural and Caspian samples in the west, and with the Altai, Tyvan and Baikal samples in the east of the range, and with the geographically marginal ones (Azov-Black Sea, Trans-Baikal and northern Khakass), the proportion of differences was within 7–13 % (Table 3). Thus, we assume that the Trans-Ural integrates the Demoiselle crane gene pool to a certain extent, possibly due to the gene flow between the European and Asian parts of the range through Central and Eastern Kazakhstan, which requires further study in the future using a set of various DNA markers and verification by independent methods. For a more complete understanding of the Demoiselle crane gene pool structure, population genetic studies need to be undertaken in Kazakhstan and Mongolia, the countries with the largest Demoiselle crane population number.

## Conclusion

So, we have demonstrated the effectiveness of using sequences of the mitochondrial cytochrome b gene, which is less variable than the Control Region, but exhibits a higher degree of interpopulation differentiation, to identify the population genetic structure of the Demoiselle crane. Based on the definition of the term “subpopulation” (interbreeding individuals with highly limited gene flow with adjacent subpopulations) and the correspondence of the intraspecific genetic differentiation data of the Demoiselle crane flyways characterized using remote tracking (Ilyashenko et al., 2021), we propose to distinguish subpopulations in the species structure, reflecting their breeding and wintering grounds in their names: 1) Azov-Black Sea/Chadian (Azov-Black Sea region – Chad); 2) Caspian/ Sudanese (Caspian region, Trans-Volga, Cis-Urals – Sudan); 3) Trans-Ural/Indian (East of the Orenburg Region, Northern Kazakhstan and presumably the Chelyabinsk Region – India); 4) South Siberian/Indian (Altai, Khakassia, Tyva – India); 5) Baikal/Indian (Buryatia, Irkutsk Region – India) and 6) Trans-Baikal/Indian (Zabaikalsky Krai – India) (Fig. 1b, c)

The obtained results create a basis for monitoring the genetic diversity of the Demoiselle crane and developing a scientific justification for its protection measures at the level of species, subpopulations and local breeding groups. Further comprehensive studies (remote tracking and molecular genetic analysis) in other parts of the range will contribute to a more complete understanding of the factors of isolation and integration of the gene pool of this crane species.

## Conflict of interest

The authors declare no conflict of interest.
